# Pivot, persevere, progress: Reinventing yourself in electrophysiology and beyond

**DOI:** 10.1016/j.hrcr.2025.09.010

**Published:** 2025-11-14

**Authors:** Jagmeet P. Singh

**Affiliations:** Division of Cardiology, Harvard Medical School, Massachusetts General Hospital, Boston, Massachusetts

My father often reminded me: *“You are not born to profess just one profession.”* It took me a long time to understand what he meant. But over the years, I have realized he was right. It is not only acceptable—it is essential—to change lanes, to reinvent yourself, and to recognize when the enthusiasm for something that once defined you begins to fade. The key is honesty with yourself. Of course, this comes with risk: stepping outside your comfort zone, embracing uncertainty, and seizing opportunities that test resilience.

For me, cardiac electrophysiology (EP) became that platform. It is a field uniquely diverse—deep in science, rich in innovation, and vast in unexplored territory. EP gave me the freedom to pivot, to reinvent, and to become who I truly am. Carl Jung once said, *“The greatest privilege of a lifetime is to become who you truly are.”* With humility, I can say EP gave me that privilege. It has engaged my surgical instincts, satisfied my curiosity for practice-changing research, opened doors to teach around the world, and offered the chance to lead. It even gave me a creative outlet through writing and speaking.

## Early years: Learning to adapt

My journey began in India, as the son of an army officer. Growing up, I changed schools 6 or 7 times, which meant I learned to adapt quickly. That adaptability became one of the most valuable skills I carried into medicine.

I completed medical school and early training in Pune and Bombay. During those years, I discovered research and was fortunate to publish early work and receive a scholarship to Oxford. At Oxford, I began with a basic science project, staring down single cardiac myocytes through a microscope. It did not take long for me to realize that was not my strength. I lacked the patience and perseverance for that kind of science.

But instead of walking away, I changed course. I reshaped my thesis into a clinical and translational project that intersected with electrocardiogram signal processing, QT dynamics, and risk stratification. That pivot not only led to a PhD in 3 years but also laid the foundation for my future in EP.

## Expanding horizons

Next came Massachusetts. I joined the Framingham Heart Study as a research fellow, then entered Mass General as an intern. I stayed to complete residency, cardiology fellowship, and subspecialty training in EP. I had come to the United States (US) with the intent to build an academic career centered on device innovation, and I stayed true to that vision.

Starting as junior faculty in 2003, I found myself at the dawn of cardiac resynchronization therapy. It was a new frontier—merging EP and heart failure, demanding technical skill, and offering opportunities for innovation. I hitched my wagon to this rising wave, helping to define the field through novel pacing techniques and individualized care.

My fascination with device sensors expanded into risk stratification research, and the creation of one of the first multidisciplinary heart failure clinics. Those experiences taught me leadership in a very real way—not from textbooks, but from building teams to take care of fragile patients who needed everyone working together.

## Reinvention in digital health

As I took on more leadership roles, I began to see barriers to progress in daily practice—bureaucracy, inefficiency, and inertia. I realized we needed to rethink the way we deliver care. That led me into digital health, a space in which I had never formally trained. Beyond taking courses at Massachusetts Institute of Technology, I dabbled in entrepreneurship, launching and closely working with start-ups, that then allowed me to think big and work swiftly.

I reeducated myself, spoke with engineers, designers, policy makers, and wrote *Future Care*, a book exploring sensors, artificial intelligence, and sustainable workflows. The book was my way of taking the conversation outside hospitals and into patients’ homes. Instead of building another skyscraper, I wanted to help change the skyline. To my surprise, the impact was far beyond what I had imagined.

## A global education

One of the gifts of my career has been working across 3 continents. Each move—from India to the United Kingdom to the US—came with challenges, but also enormous growth. Training in different health care systems taught me cultural humility, and reminded me that innovation is not just about technology—context matters.

A therapy that works in Boston may look very different in Bombay. For trainees, I cannot emphasize enough the value of global exposure. Seek collaborations, exchanges, or even virtual networks. The future of EP is global, and the greatest breakthroughs will come from blending diverse experiences and perspectives.

## Leadership and mentorship

As I took on leadership roles, I began to understand that progress in medicine is rarely an individual sport. True leadership is about creating platforms for others to thrive—whether that means building teams, shaping new programs, or mentoring the next generation.

Mentorship has been a constant theme in my career. I have been fortunate to benefit from outstanding mentors at every stage, and I have tried to pay that forward. Mentorship in EP is not just about teaching procedural skill—it is about modeling resilience, curiosity, and humility. For trainees, my advice is to seek mentors who complement each other, because no single person can provide all the answers. And remember, as you grow, you too must become a mentor.

## Lessons along the way

If I could distill my journey into a few lessons for those starting out, they would be these:

1. **Be patient.** The path may zigzag across continents, and you may fail more than once. That is fine. If you love what you do, it will reward you.
2. **Be honest with yourself.** Interests evolve. Stories change. Mine did—several times. Do not be afraid to pivot.
3. **Say yes to opportunities.** They rarely arrive perfectly packaged. Whatever lands on your desk, do it well. Doors you do not expect will open.
4. **Start with the problem, not the solution.** Too many invent a tool and then look for a use. In medicine, it works best the other way around.
5. **Find the right mentors.** Often, you will need more than one. Choose people who complement each other. Progress is a climb: crawl, walk, run—only then fly.
6. **Stay humble.** My mother’s reminder. Success is sweeter when shared with colleagues and friends. Growth is not a zero-sum game.

## Closing thoughts

To those beginning their careers in EP: I wish you fast lanes that keep you excited, but also slow enough to let you savor the journey. Reinvention is not a detour—it is the journey itself.

EP is more than catheters and devices. It is a field that demands creativity, resilience, and the courage to redefine yourself again and again. Your career will not unfold as a straight line—mine certainly did not. But that is where the richness lies.

As you move forward, embrace uncertainty, take risks, and stay open to reinvention. Because in EP, as in life, the true measure of success is not how fast you get to the finish line, but how fully you grow along the way.

## Disclosures

Consulting/Lectures: Abbott Inc, Biotronik Inc, Boston Scientific, Cardiologs Inc, CVRx Inc, Carelog Inc, CoreDio Inc, EBR Inc, Impulse Dynamics, Medtronic Inc, Medscape Inc, Microport Inc, Myochron, Notal Vision, Orchestra BioMed Inc, Oura Inc, Phillips Inc, Sanofi Inc, SmartCardia Inc, and Vektor Medical Inc. Research: Heart Rhythm Society, American College of Cardiology, Abbott Inc, Biotronik Inc, Boston Scientific, Cardiologs Inc, EBR Inc, Medtronic Inc, Sanofi Inc, and SentiAR Inc. Equity/Board: Carelog Inc, CoreDio Inc, Circadian Inc, Oura Inc, and SmartCardia Inc.

